# Mechanisms underlying response and resistance to immune checkpoint blockade in cancer immunotherapy

**DOI:** 10.3389/fonc.2023.1233376

**Published:** 2023-07-28

**Authors:** Junghwa Lee, Eui Ho Kim

**Affiliations:** Viral Immunology Laboratory, Institut Pasteur Korea, Seongnam, Republic of Korea

**Keywords:** cancer, immune checkpoint blockade, PD-1, CTLA-4, response, resistance

## Abstract

Cancer immunotherapies targeting immune checkpoint pathways, such as programmed cell death-1 (PD-1)/programmed cell death ligand-1 (PD-L1) and cytotoxic T-lymphocyte-associated antigen-4 (CTLA-4), have achieved unprecedented therapeutic success in treating various types of cancer. The prominent and persistent clinical responses to immune checkpoint blockade (ICB) therapy are currently constrained to a subset of patients. Owing to discrete individual tumor and immune heterogeneity, most patients fail to benefit from ICB treatment, demonstrating either primary or acquired resistance. A thorough comprehension of the mechanisms restricting the efficacy of immune checkpoint inhibitors (ICIs) is required to extend their clinical applicability to a broader spectrum of patients and cancer types. Numerous studies are presently investigating potential prognostic markers of responsiveness, the complex dynamics underlying the therapeutic and adverse effects of ICB, and tumor immune evasion throughout the course of immunotherapy. In this article, we have reviewed the extant literature elucidating the mechanisms underlying the response and resistance to ICB, with a particular emphasis on PD-1 and CTLA-4 pathway blockade in the context of anti-tumor immunity. Furthermore, we aimed to explore potential approaches to overcome cancer therapeutic resistance and develop a rational design for more personalized ICB-based combinational regimens.

## 1 Introduction

Prolonged antigenic stimulation and the ensuing inflammation in tumor microenvironments (TME) lead to immunosuppression, which results in different states of T cell exhaustion. Continuous exposure to antigens causes antigen-specific T cells to lose their effector functions and proliferative capacity and acquire multiple inhibitory receptors. Therefore, restoring the functionality of tumor-specific T cells is a crucial objective of cancer immunotherapy. Immune checkpoint-targeting interventions have demonstrated significant anti-tumor efficacy; nonetheless, ICB has elicited therapeutic responses only in a subset of patients (1). The molecular mechanisms associated with the PD-1 or CTLA-4 pathway-mediated negative regulation of T cell responses and the alterations in PD-1 or CTLA-4 signaling upon blocking their interactions to revive exhausted T cells warrant further comprehensive elucidation. Moreover, it is essential to identify the predictive biomarkers of ICB-mediated responsiveness for designing personalized therapies.

## 2 Biological mechanisms and clinical effects of ICB

### 2.1 CTLA-4 pathway blockade

**Figure 1:**
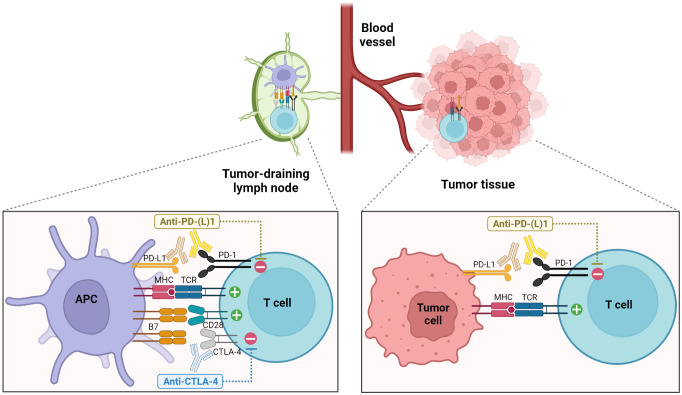
Enhancement of anti-tumor T cell responses through the blockade of PD-1 and CTLA-4 pathways. Blockade of CTLA-4 primarily acts during the priming phase in secondary lymphoid organs, promoting the interactions between T cells and APCs and leading to increased T cell proliferation and activation. On the other hand, PD-1 pathway blockade elicits the functional restoration of exhausted T cells in the TME as well as the enhanced priming of tumor-specific T cells in the TDLNs (1, 5).

CTLA-4 is a co-inhibitory receptor primarily expressed by T cells. Intracellular CTLA-4 is translocated to the cell surface following the recognition of cognate antigens early after T cell activation where they compete with CD28 for interacting with B7 molecules on antigen-presenting cells (APCs), exhibiting a higher affinity (2). Subsequently, this interaction delivers negative signaling to T cells, leading to the attenuation of T cell proliferation, activation, and overall function (3, 4) (**Figure 1**). CTLA-4 is also constitutively expressed in regulatory T cells (Tregs) (6–8). A close association between CTLA-4 and the maintenance and function of Tregs has been demonstrated. CTLA-4-knockout mice suffer from lymphoproliferative disorders and early fatality (9, 10). Treatment with an anti-CTLA-4 antibody effected a pronounced and persistent regression of established tumors in syngeneic mouse models (11). Subsequently, clinical trials evaluated fully human anti-CTLA-4 blocking antibodies in patients with advanced cancers, revealing a durable response in a certain proportion of patients, irrespective of the relatively high frequency of associated toxicity. In an initial clinical trial where an anti-CTLA-4 blocking antibody was administered to patients with metastatic melanoma in combination with peptide vaccines containing melanoma-associated antigens, 43% of patients (6 out of 14) experienced grade III/IV autoimmunity affecting multiple organs (12). In 2011, ipilimumab was licensed as the first ICI for the treatment of metastatic melanoma (13, 14).

The anti-tumor effects of CTLA-4 blockade have been shown to be accompanied by an increase in effector T cells and a decrease in Tregs, thereby leading to an elevated ratio of intratumoral effector T cells to Tregs in mouse tumor models (15, 16). These findings have also been observed in studies involving patients with melanoma who received ipilimumab, both in peripheral blood and tumor tissues (17, 18). Additionally, a recent study demonstrated that local administration of tremelimumab resulted in reduced Tregs in the sentinel lymph nodes and peripheral blood of melanoma patients (19). However, it is noteworthy to mention that contrasting results have been reported in multiple studies where increased or maintained levels of Tregs in peripheral blood and tumor-infiltrating lymphocytes (TILs) of cancer patients were indicated following anti-CTLA-4 therapy (20), suggesting variable outcomes regarding the impact of CTLA-4 blockade on Tregs in relation to human tumors.

### 2.2 PD-1 pathway blockade

PD-1, an inhibitory receptor of the CD28 family, predominantly regulates T cell exhaustion and suppresses T cell anti-tumor effector functions under persistent antigenic stimulation. PD-1 is expressed in various hematopoietic cells and binds to two members of the B7 family of ligands— PD-L1 and PD-L2 (21–24). PD-L1 is widely expressed in both hematopoietic and non-hematopoietic cells. Contrarily, PD-L2 expression is more restricted to professional APCs and a specific subset of B cells. PD-L1 and PD-L2 expression is induced by inflammatory cytokines such as interferons (25). Activation-driven T-cell receptor (TCR) signaling induces the expression of PD-1, which is further upregulated by cytokines. Engagement of PD-1 with its ligands recruits the tyrosine phosphatase 2-containing SH2-domain (SHP-2) that inactivates proximal effector molecules, namely zeta-chain-associated protein kinase-70 and phosphoinositide 3-kinase (PI3K), thereby attenuating TCR- and CD28-mediated signaling (26–28). The immune-regulatory function of PD-1 is evidenced through the development of an autoimmune-like phenotype in mice lacking PD-1 with a delayed onset compared with CTLA-4-knockout mice (29, 30). In the TME, continuous antigenic stimulation leads to sustained high-levels of PD-1 expression on T cells. Additionally, inflammatory cytokines such as interferon-γ (IFN-γ) induce upregulation of PD-L1 on tumor cells, facilitating the PD-1/PD-L1 interaction and ultimately contributing to T cell exhaustion. The PD-1 pathway restrains the interactions between tumor-infiltrating PD-1+ T cells and other immune cells or tumor cells expressing PD-1 ligands in the TME as well as tumor antigen presentation by APCs to tumor-specific T cells during priming in the tumor-draining lymph nodes (TDLNs) (**Figure 1**). *In vivo* studies investigating the treatment with anti-PD-1 or PD-L1 antibodies have reported delayed tumor progression and enhanced viral control accompanied by the reinvigoration of virus-specific T cells in syngeneic murine tumor models and chronic viral infection models, respectively (31, 32). These preclinical studies have prompted the development of human PD-1- and PD-L1-targeting antibodies and the initiation of clinical trials involving patients presenting with advanced malignancies refractory to conventional treatment (33–36). In 2014, the Food and Drug Administration (FDA) authorized a PD-1-targeting ICI for treating patients with advanced melanoma. Currently, multiple FDA-approved PD-1-targeting ICIs (pembrolizumab, nivolumab, cemiplimab, dostarlimab, and retifanlimab) or PD-L1-targeting ICIs (atezolizumab, durvalumab, and avelumab) are available for remedying various types of cancer. Additionally, PD-1 blockade therapy exhibits a relatively favorable toxicity profile in comparison to CTLA-4 blockade therapy. Furthermore, the distinct mechanisms of action of PD-1 and CTLA-4 pathway blockade, along with the observed synergistic anti-tumor effects of combined treatment in preclinical models (37–39), led to the clinical evaluation of these two ICIs for cancer patients, demonstrating an enhanced clinical response (40). Subsequently, the combination therapy of nivolumab and ipilimumab was approved in 2016 for the treatment of metastatic melanoma patients.

Recent findings on heterogeneous exhausted CD8 T cell subsets with varying qualities have revealed that PD-1 blockade therapy acts primarily on progenitor exhausted CD8 T cells, promoting their proliferation and differentiation into more terminally exhausted CD8 T cells with effector functions (41–44). Considering the significance of progenitor exhausted CD8 T cells in sustaining the exhausted CD8 T-cell pool, it is crucial to identify the mechanisms regulating their maintenance during chronic antigenic stimulation. Moreover, recent discoveries have explicitly demonstrated the prominence of anatomical effector sites in PD-1 pathway blockade. FTY720 treatment-mediated prevention of T cell egress via secondary lymphoid organs in murine tumor models abolished the therapeutic effect of anti-PD-L1 antibodies (45, 46). These findings suggest that the restoration of exhausted T cell function by anti-PD-L1 antibodies could be more closely associated with immune cells located in TDLNs than with those in the TME. Compared with systemic treatments, local administration of anti-PD-L1 antibodies targeting TDLNs elicited superior anti-tumor therapeutic effects, and PD-L1+ conventional dendritic cells (DCs), rather than other myeloid cells, were the primary targets of PD-L1 inhibition (47–49). Studies conducted on cancer patients have provided that anti-PD-L1 antibodies exert a therapeutic effect through the expansion of pre-existing tumor-specific T cells and the infiltration of new clonotype T cells, thereby effecting clonal replacement (1, 50, 51). PD-1 is also highly expressed in tumor-infiltrating Tregs (52). Whether cell-intrinsic PD-1 exerts a stimulatory or inhibitory effect in Tregs remains controversial; nonetheless, a recent study demonstrated that PD-1 deletion in Tregs impairs their proliferation and inhibitory function in the TME (53).

## 3 Resistance Mechanisms of ICB

Despite the unprecedented durable outcomes achieved with cancer immunotherapies, many patients may present with either primary resistance, which indicates irresponsiveness to ICB, or acquired resistance, which develops in patients who initially respond to treatment, and eventually deems the tumor refractory. Various mechanisms regulating the therapeutic resistance to ICBs have been elucidated and implicated in multiple cancer indications (**Table 1**).

**Table 1:** Mechanisms of primary and acquired resistance to ICB.

Tumor cell – intrinsic	◼ Low levels of TMB / neoantigens ◼ Defects in antigen presentation - loss of *B2M* - loss of HLA - overexpression of *MEX3B* ◼ Activation of oncogenic signaling - activation of PI3K/loss of PTEN - activation of β-catenin ◼ Defects in IFN-γ signaling - mutations of genes encoding *JAK1, JAK2, IFNGR1, IFNGR2, IRF1* ◼ Epigenetic alterations	(54–62) (59, 63–66) (62, 67–69) (64, 70, 71) (72–75)
Tumor cell – extrinsic	◼ Suppressive immune cells - MDSC, TAM, Treg ◼ Gut microbiome ◼ Upregulation of additional immune checkpoint molecules - TIM-3, VISTA, LAG-3, TIGIT, 2B4 ◼ Other: inhibitory cytokines, growth factors	(76–80) (81–84) (66, 85, 86) (87–89)

### 3.1 Tumor-intrinsic mechanisms for primary resistance to ICB

#### 3.1.1 Tumor mutational burden/antigen presentation

To generate anti-tumor T cell responses, the first step is the recognition of tumor antigens that are distinct from self-antigens by T cells. Although tumor mutational burden **(**TMB) alone is not sufficient to prognosticate the benefit of treatment, prior research has depicted a relationship between the neoantigen load arising from tumor-specific mutations and the clinical activity of ICB. A significant correlation has been established between the high TMB and improved clinical outcomes from anti-PD-1/PD-L1 therapy across diverse types of cancer (54–56). Similarly, a higher mutational burden and neoantigen load were associated with enhanced clinical responses and survival after anti-CTLA-4 therapy in patients with melanoma (57, 58). Furthermore, patients with mismatch repair-deficient/high microsatellite instability tumors, which generate a high level of somatic mutations, are more likely to benefit from anti-PD-1 therapy in multiple cancer types (59, 60). Therefore, these data indicate that low levels of available neoantigens, resulting from a lack of sufficient tumor mutational load, can influence primary resistance to ICB. Besides insufficient tumor antigenicity, defective antigen presentation may be associated with tumor resistance to ICB by rendering tumor cells incapable of being recognized and killed by T cells. Consequently, the expression of *MEX3B* mRNA in the pretreated melanoma tumors, which downregulated human leukocyte antigen (HLA)-A expression on tumor cells by binding to the 3’ untranslated region, was noted to be higher in non-responders than responders to anti-PD-1 therapy (63).

#### 3.1.2 Oncogenic signaling

Tumor-intrinsic oncogenic signaling plays a role in ICB resistance. Activation of the tumor-intrinsic PI3K pathway via phosphatase and tensin homolog (PTEN) loss is inversely associated with the efficacy of anti-PD-1 therapy. Patients with metastatic melanoma who received anti-PD-1 therapy have shown that PTEN-expressing tumors were associated with an improved objective response compared with tumors with PTEN deficiency (67). The Cancer Genome Atlas (TCGA) analysis of melanoma demonstrated that a higher frequency of PTEN deletion and loss of function was observed in non-T cell-inflamed tissues than in T cell-inflamed tissues. A mouse melanoma model has shown that tumor-intrinsic PTEN loss results in reduced T-cell tumor infiltration accompanied by elevated expression of inhibitory cytokines and inhibition of autophagy, with subsequent escape from T-cell killing. Furthermore, PI3K inhibition combined with anti-PD-1 therapy enhanced the therapeutic effects of ICB, leading to further tumor regression and improved survival in tumor-bearing mice. In another study on metastatic cutaneous melanoma patients, the activation of the β-catenin signaling was one of the primary features of non-T cell-inflamed tumors compared with T cell-inflamed tumors (68). In a preclinical melanoma model, tumor-intrinsic β-catenin activation was shown to impair the infiltration of T cells and CD103+ DCs, which is related to reduced CCL4 secretion from tumors and leads to therapeutic resistance to the combined PD-1 and CTLA-4 blockade. These data indicate that specific oncogenic signals in tumors can cause immune exclusion, contributing to primary resistance to ICB.

#### 3.1.3 IFN-γ signaling

In the TME, T cells produce cytokines, such as IFN-γ, upon recognizing specific tumor antigens. IFN-γ serves as a strong inducer of PD-L1 expression, enabling the tumor to evade the anti-tumor immune response. The therapeutic effects of PD-1 pathway blockade involve the disruption of adaptive immune resistance that relies on pre-existing anti-tumor immunity (90). The presence of an IFN-γ signature from the tumor has been proposed as an indicator of responsiveness to anti-PD-1 therapy (91, 92). IFN-γ has both stimulatory and inhibitory effects on the anti-tumor immune response (93). In the TME, IFN-γ promotes anti-tumor immunity not only by activating and recruiting immune cells but also by increasing the expression of major histocompatibility complex (MHC) molecules by tumor cells and directly exerting an anti-proliferative and pro-apoptotic effect on tumor cells. Considering the critical role of IFN-γ in anti-tumor immunity, tumor-intrinsic alterations that disrupt the IFN-γ pathway could interfere with the efficacy of ICB therapy. Melanoma tumors from non-responders to ipilimumab have displayed a higher frequency of mutations such as copy-number alterations in IFN-γ pathway genes, including *IFNGR1*, *IRF1*, and *JAK2*, than that from the responders (70). Consistently, tumor-intrinsic *IFNGR1* deficiency was associated with reduced therapeutic effects of CTLA-4 blockade, resulting in accelerated tumor growth and reduced survival of B16/BL6-bearing mice, indicating an association between the lack of tumor-intrinsic IFN-γ signaling and primary resistance to CTLA-4 blockade. Another study has demonstrated that human melanoma cell lines harboring loss-of-function mutations of *JAK2* failed to upregulate PD-L1 in response to IFN-γ exposure (71), implying that patients with defective *JAK2*-harboring tumors would result in the absence of inducible PD-L1 expression and be unlikely to benefit from anti-PD-1 therapy, representing primary resistance.

#### 3.1.4 Epigenetic alterations

Epigenetic modifications occurring in tumor cells may contribute to immune evasion and resistance to ICIs. In an ovarian cancer model, mice harboring tumors deficient in *ARID1A*, a component of the switch/sucrose non-fermentable (SWI/SNF) chromatin remodeling complex, exhibited increased TMB and impaired mismatch repair capacity, and notably demonstrated reduced tumor growth and improved survival in response to anti-PD-1 therapy compared to those with control tumors (72). Similarly, mouse melanoma lacking *Pbrm1*, another subunit of the SWI/SNF complex, displayed increased susceptibility to combined PD-1 and CTLA-4 blockade than control tumors (73). In line with these findings, a study involving renal cell carcinoma patients receiving PD-1 pathway blockade monotherapy or combination therapy with CTLA-4 blockade showed that tumors with loss of function mutations in *Pbrm1* correlated with clinical benefits from the treatment (74). Additionally, epigenetic modifying agents, such as DNA methyltransferase inhibitors and histone deacetylases inhibitors, have the potential to significantly impact genes associated with tumor antigenicity, cytokine and chemokine production, as well as oncogenic or tumor-suppressive signaling (75, 94). These agents have been proposed as potential therapeutic strategies for restoring anti-tumor immunity and the clinical efficacy of combing these inhibitors with ICB has been evaluated.

### 3.2 Tumor-extrinsic mechanisms for primary resistance to ICB

Suppressive immune cells have been implicated as mediating ICB resistance. Myeloid-derived suppressor cells (MDSCs) play a role in promoting tumor growth, angiogenesis, and metastasis, as well as inhibiting anti-tumor immunity (95). A study on patients with metastatic melanoma receiving ipilimumab showed that responders had a significantly lower frequency of circulating monocytic MDSCs than non-responders (76). In addition, treatment with the selective PI3K inhibitor to inhibit MDSCs, in combination with ICIs enhanced tumor regression and survival than ICB blockade alone in multiple tumor models, indicating that reprograming MDSCs could stimulate the response to ICB (77, 78). Tumor*-*associated macrophages (TAMs) exert immunosuppressive effects by upregulating the molecules involved in the inhibitory pathway, secreting regulatory cytokines, and promoting tumor invasion and metastasis (96). An association between a higher frequency of TAMs and a poor prognosis in human cancer has been cataloged for several types of cancer. Macrophage depletion has demonstrated reduced tumor growth in various preclinical models. In a study conducted on murine pancreatic cancer, blocking the colony-stimulating factor-1 receptor to deplete macrophages led to substantial tumor regression when combined with PD-1 and CTLA-4 blockade, which otherwise had no significant anti-tumor effects, indicating the role of TAMs in the therapeutic effects of ICB (79). Numerous studies have reported Treg infiltration in human tumors (20). Preclinical models have demonstrated that depletion of Tregs in the TME can enhance anti-tumor immunity. In patients receiving nivolumab, a reduced frequency of peripheral blood Tregs was observed in responders and patients with stable diseases whereas an increased Treg frequency was noted in non-responders (80). These results indicate the potential of reprogramming suppressive immune cells to enhance the response to ICB.

The gut microbiome has been reported to influence and modulate responses to ICB therapy. Considerable variations in the diversity and composition of the gut microbiome have been observed between responders and non-responder melanoma patients receiving anti-PD-1 therapy (81). Responders had a favorable gut microbiome that promotes anti-tumor immunity. Another study showed correlations between clinical responses to anti-PD-1 therapy and *Akkermansia muciniphila* in the gut microbiome. Oral supplementation with *A. muciniphila* following fecal microbiota transfer from non-responders resulted in the restoration of anti-PD-1 efficacy (82). In addition, antibiotic treatment reduced the clinical benefits of anti-PD-1 therapy in patients with advanced cancer (83). Similarly, the efficacy of anti-CTLA-4 therapy was associated with specific bacterial species, and antibiotic-treated or germ-free mice did not respond to CTLA-4 blockade (84). These data suggest that the microbiome could be a critical factor in causing resistance and implicate the manipulation of the microbiome to augment the efficacy of ICB.

Immunosuppressive cytokines such as transforming growth factor-β (TGF-β) contribute to ICB resistance. TGF-β signaling in fibroblasts was associated with CD8 T cell exclusion from the tumor parenchyma and resistance to anti-PD-1 therapy in patients with metastatic urothelial cancer (87). A preclinical study has shown that the combination of PD-L1 and TGF-β blocking antibodies resulted in increased infiltration of T cells into the tumor and a significant reduction in tumor growth compared with either blockade alone. Additionally, growth factors such as vascular endothelial growth factor (VEGF) have been found to correlate with resistance to anti-PD-1 therapy in melanoma patients (88). The combined blockade targeting PD-1 and VEGF induced an improved anti-tumor effect in patients with renal cell carcinoma (89). Other inhibitory molecules and pathways have been implicated in limiting the efficacy of ICB, and further study will be required to verify the relationship with ICI resistance.

### 3.3 Mechanisms for acquired resistance to ICB

Cancer immunoediting involves three distinct phases: elimination, equilibrium, and escape. It reflects the intricate interplay between the tumor and the immune system during tumor progression, and these dynamics can also occur in response to immunotherapy (97). When the tumor reaches a state where it can evade the anti-tumor immune response and the immunotherapy fails to adequately counteract tumor-mediated immune suppression, the tumor acquires resistance to the treatment.

It has been reported that loss of neoantigens could be associated with acquired resistance to ICB. Examination of the emergence of acquired resistance in patients with non-small cell lung cancer who initially responded to PD-1 blockade or combined PD-1 and CTLA-4 blockade therapy revealed a decrease in mutation-associated neoantigens in resistant tumors compared with pretreatment tumor samples (61). This loss of neoantigens occurred due to subclonal elimination and chromosomal deletion and it was associated with decreased T-cell clonotypes. Additionally, another study reported a reduction in neoantigen expression from patients with uterine leiomyosarcoma who developed acquired resistance to anti-PD-1 therapy (62).

β-2-microglobulin (B2M), a subunit of the MHC class I complex, plays an essential role in the assembly and trafficking of MHC class I molecules and antigen presentation. Accordingly, *B2M* deficiency can lead to immune evasion by tumors. Genetic alterations in *B2M* have been reported in several types of cancer that have developed acquired resistance to ICB. Whole-exosome sequencing assessment of baseline and relapsed tumors from four patients with melanoma who developed resistance to anti-PD-1 therapy after the initial response identified an acquired homozygous truncating mutation in the gene encoding *B2M* at relapse in one patient (64). Another longitudinal study examining five melanoma patients with acquired resistance to ICB reported a patient with two frameshift mutations in *B2M* and a second patient harboring a single *B2M* loss with two frameshift mutations during progression, which resulted in a significant reduction in tumor expression of B2M and a lack of HLA class I protein expression on the outer membrane (65). In addition, a newly generated truncating *B2M* mutation was reported in two patients with brain metastasis among five patients with advanced mismatch repair-deficient cancers who demonstrated acquired resistance to anti-PD-1 therapy (59). A study of lung cancer with 14 ICB-resistant tumor samples also revealed the homozygous loss of *B2M* in a patient who acquired resistance to combined anti-PD-L1 and anti-CTLA-4 therapy (66). Therefore, these data indicate that impaired tumor antigen presentation could also be a mechanism of acquired resistance that develops among responders to ICB.

Tumors exhibiting PTEN loss have also been observed in patients who experienced relapse after an initial clinical response to ICB. In the case of uterine leiomyosarcoma patients who developed acquired resistance following an initial response to anti-PD-1 therapy, biallelic PTEN loss was detected (62). Similarly, a separate study identified a patient with metastatic melanoma who demonstrated a partial response to the combination of anti-PD-1 and anti-CTLA-4 therapy but later developed treatment-resistant metastasis with acquired PTEN loss (69).

Defective IFN-γ signaling has also been documented in ICB-resistant tumors upon relapse. A study has depicted that loss-of-function mutations in *JAK1* or *JAK2* were found in two of four melanoma patients presenting with acquired resistance to anti-PD-1 therapy (64). The cell lines established from relapsed tumors of the patient harboring the *JAK2* mutation exhibited an absence of JAK2 protein and loss of sensitivity to IFN-γ signaling, concurrently with reduced expression of IFN-γ-responsive PD-L1 and MHC I, in contrast to those derived from the baseline tumors.

It has been documented that in the TME at the time of acquired resistance, there is an upregulation of other immune checkpoint molecules, which may have non-redundant roles. This suggests the existence of potential resistance mechanisms and implies that combined blockade targeting multiple immune checkpoints could result in synergistic therapeutic efficacy. Notably, an analysis of two lung cancer patients who experienced a partial response to anti-PD-1 therapy but eventually progressed revealed upregulated expression of T cell immunoglobulin and mucin domain-containing protein 3 (TIM-3) on TILs, to which anti-PD-1 antibodies were bound, at the time of resistance (85). In a mouse model of lung cancer, elevated expression of TIM-3 was similarly observed on anti-PD-1 antibody-bound T cells, and subsequent blockade of TIM-3 improved overall survival than PD-1 blockade alone. Additionally, a separate report found that 67% of melanoma patients who developed resistance following the initial response to anti-PD-1 monotherapy or a combination of anti-PD-1 and anti-CTLA-4 therapy exhibited increased numbers of intratumoral V-domain Ig suppressor of T cell activation (VISTA)+ lymphocytes upon disease progression compared with pretreatment levels (86). Additionally, an upregulation of the expression of multiple inhibitory receptor genes, including PD-1, CTLA-4, TIM-3, lymphocyte activation gene-3 (LAG-3), T cell immunoreceptor with Ig and ITIM domains (TIGIT), and 2B4, was observed in lung cancer samples that developed resistance to PD-1 pathway blockade therapy compared with their corresponding pretreatment samples (66).

## 4 Discussion

Numerous factors and mechanisms contribute to primary and acquired ICB resistance. Specific immunomodulatory strategies are envisaged to induce cancer immunoediting and consequent immune escape. Clinical trials are actively assessing the efficacy of combination cancer therapies involving multiple ICIs and other therapeutic interventions. Therefore, endeavors directed toward comprehensively elucidating the response, limitations, and irresponsiveness to ICB can provide insight into identifying prognostic biomarkers of ICB-mediated response to formulate personalized combinational therapeutic regimens. Consequently, this would enhance clinical outcomes for a broader spectrum of cancer patients.

## Author contributions

JL researched and drafted the manuscript. EHK supervised the content. All authors contributed to the article and approved the submitted version.
